# Anesthetic-Induced Oxidative Stress and Potential Protection

**DOI:** 10.1100/tsw.2010.118

**Published:** 2010-07-20

**Authors:** Cheng Wang, Xuan Zhang, Fang Liu, Merle G. Paule, William Slikker, Jr.

**Affiliations:** National Center for Toxicological Research, U.S. Food and Drug Administration, Jefferson, AR, USA

**Keywords:** N-methyl-D-aspartate (NMDA) receptors, reactive oxygen species (ROS), neurodegeneration, protective agents

## Abstract

Prolonged exposure of developing mammals to general anesthetics affects the N-methyl-D-aspartate (NMDA)–type glutamate or γ-aminobutyric acid (GABA) receptor systems and enhances neuronal toxicity. Stimulation of immature neurons by NMDA antagonists or GABA agonists is thought to increase overall nervous system excitability and may contribute to abnormal neuronal cell death during development. Although the precise mechanisms by which NMDA antagonists or GABA agonists cause neuronal cell death are still not completely understood, up-regulation of the NMDA receptor subunit NR1 may be an initiative factor in neuronal cell death. It is increasingly apparent that mitochondria lie at the center of the cell death regulation process. Evidence for the role of oxidative stress in anesthetic-induced neurotoxicity has been generated in studies that apply oxidative stress blockers. Prevention of neuronal death by catalase and superoxide dismutase *in vitro*, or by M40403 (superoxide dismutase mimetic) *in vivo*, supports the contention that the involvement of reactive oxygen species (ROS) and the nature of neuronal cell death in rodents is mainly apoptotic. However, more evidence is necessary to in order verify the role of the NMDA receptor subunit NR1 and ROS in anesthetic-induced neurodegeneration.

## INTRODUCTION

Lines of evidence have indicated that activation of *N*-methyl-D-aspartate (NMDA) or γ-aminobutyric acid (GABA) receptors caused neuronal cell death[1,2,3,4,5,6]. Although neuronal apoptosis can be the final result of anesthetic-induced toxicity, the pathways leading to apoptosis are not completely understood. It is increasingly apparent that mitochondria lie at the center of the cell death regulation process. Continuous exposure of developing brains to general anesthetics may cause selective cell death by a mechanism that involves a dysregulation of NMDA receptor subunits[7,8,9], accompanied by loss of mitochondrial membrane potential, alterations of calcium homeostasis, and subsequent free radical formation. In addition, several factors may contribute to the topographic differences in anesthetic-induced neurodegeneration, such as animal species, developmental stage at the time of drug exposure, doses and duration of anesthesia exposure, etc. The role of oxidative stress in anesthetic-induced neurotoxicity has been indicated in studies that apply oxidative stress blockers, including L-carnitine (mitochondrial protector) and melatonin *in vivo*, and specific antioxidants *in vitro*, including the superoxide dismutase mimetic, M40403, and the nitric oxide synthase (NOS) inhibitor, 7-nitroindazole. Recent gene expression assessments indicate that genes along the oxidative stress pathway are altered by anesthetic treatment of developing animals. Together, the application of omics approaches along with traditional toxicological end points suggests that the susceptibility of the developing brain to anesthetics is mediated by oxidative stress.

## REACTIVE OXYGEN SPECIES INVOLVEMENT IN ANESTHETIC-INDUCED NEURODEGENERATION AND POTENTIAL PROTECTIVE AGENTS FOR OXIDATIVE STRESS

It is proposed that the administration of general anesthetics during critical developmental periods will result in a dose-related increase in neurotoxicity and loss of neurons[8,9,10,11]. Polymers of α-2,8-linked sialic acid neural cell adhesion molecule (PSA-NCAM), a neuronal specific marker, is formed by the enzymatic transfer of large, negatively charged carbohydrate polymers of PSA to the fifth immunoglobulin domain of the NCAM molecule. To determine whether the expression levels of PSA-NCAM correlate with inhaled anesthetic-induced cell death, Western blot analysis for PSA-NCAM was performed[12]. An anesthetic combination (75% $N_2O$ + 0.55% ISO for 6 h) caused a substantial increase in caspase-3, silver, and Fluoro-Jade C staining, along with a concomitant decrease in PSA-NCAM staining. The decrease in PSA-NCAM corresponded to an approximately 45% decrease in PSA-NCAM immunoreactivity as assessed using an immunoblot assay. This decrease could be the direct result of neuronal loss induced by anesthetics, because this reduction of PSA-NCAM expression is consistent with the enhanced neurodegeneration as indicated by increased caspase-3, silver, and Fluoro-Jade C staining-positive neuronal cells.

L-carnitine is a dietary supplement and has been reported to prevent apoptotic death[13]. L-carnitine is well known to exhibit membrane modulatory effects and thereby preserves cellular membrane integrity. It plays a critical role in mitochondrial oxidation of long-chain fatty acids[14]. Thus, elevated body L-carnitine levels should enhance the capability of the mitochondrial antioxidant system and decrease the incidence of free radical–induced lipid peroxidation[15]. The fact that coadministration of L-carnitine blocked cell death, as well as the loss of PSA-NCAM immunoreactivity, further indicates that the neuronal cell death induced by an anesthetic combination in developing rats is most likely apoptosis, and reactive oxygen species (ROS) may play an important role in neuron apoptosis.

It has been reported that anesthetic drugs cause widespread and dose-dependent apoptosis in the developing rat brain[1,44,45]. The window of vulnerability to these neuronal effects of anesthetics is restricted to the period of rapid synaptogenesis, also known as the brain growth spurt. In our previous anesthetic dose response and time-course studies[43], to study the vulnerability of the immature rat brain to ketamine-induced neurotoxicity, postnatal day 7 (PND 7) rats were exposed to 5, 10, or 20 mg/kg ketamine in single or multiple subcutaneous injections with 2-h intervals, respectively. Six injections of 20 mg/kg ketamine produced the most severe neuronal damage in neocortical areas, especially in layers II and III of the frontal cortex. However, no significant effects were observed in the animals injected either one or three times with 20 mg/kg ketamine. Meanwhile, enhanced apoptotic cell death was not detected in the frontal cortex of the brains exposed to 5 or 10 mg/kg of ketamine in single or multiple injections, compared with controls. In that study, several major brain regions, including the striatum, hippocampus, thalamus, and amygdala, were examined. Repeated ketamine injections produced the most severe neuronal damage (~10-fold increase) in the frontal cortex vs. a threefold increase (or less) in the striatum, hippocampus, thalamus, and amygdala. These data suggest that the frontal cortex is the brain region most vulnerable to ketamine-induced neurotoxicity during development.

Doses of ketamine administered to children are in the range of 2–3 mg/kg (i.m.) for sedation and 5–10 mg/kg (i.m.) for induction of anesthesia. However, there is an important difference between adults and neonates. There is also a significant difference between human and research animal models, such as rodents and nonhuman primates. To understand the underlying mechanism of anesthetic-induced (e.g., ketamine) neurodegeneration, dosing regimens (six injections of 20 mg/kg ketamine) were selected for PND 7 rat pups to keep/maintain a light surgical plane of anesthesia as evidenced by lack of voluntary movement, decreased muscle tone, and minimal reaction to physical stimulation. Brain tissues from the frontal cortical levels, where the most severe neuronal damage was observed, were selected for RNA isolation and microarray analysis[16]. In support of the findings of TUNEL assay (Fig. 1), electron micrographs (Fig. 2), and previous *in vivo* data, the microarray analysis demonstrated a total of 32 genes to be involved in apoptosis. Among them, 15 genes were up-regulated and 17 genes were down-regulated (Table 1) in animals exposed to six injections of 20 mg/kg ketamine, compared with the controls[16]. The apoptosis-related genes are a group of genes that has two distinct modes of operation: proapoptosis or antiapoptosis. In response to various inducers, such as stressful stimuli or sustained elevation of intracellular calcium levels, the ultimate fate of the brain cell is determined by the roles of these apoptosis-related genes in regulating the life/death cell balance. The mechanism(s) underlying the anesthetic-induced (e.g., ketamine) neuronal cell death have not been fully elucidated. However, the microarray analysis indicated that the majority (approximately two-thirds) of up-regulated genes were proapoptotic in nature, including Agt, Clu, Gjb6, Hrk, Igfbp3, Inpp5d, Jun, Mal, Rassf5, and Txnip. As a critical gene, *Cebpb* (CCAAT/enhancer binding protein [C/EBP]), acts as a major regulator of metabolic homeostasis and is involved in many cellular processes, such as differentiation, growth, immune responses, neoplastic growth, development of the reproductive system, and pro- and antigrowth pathways[17].

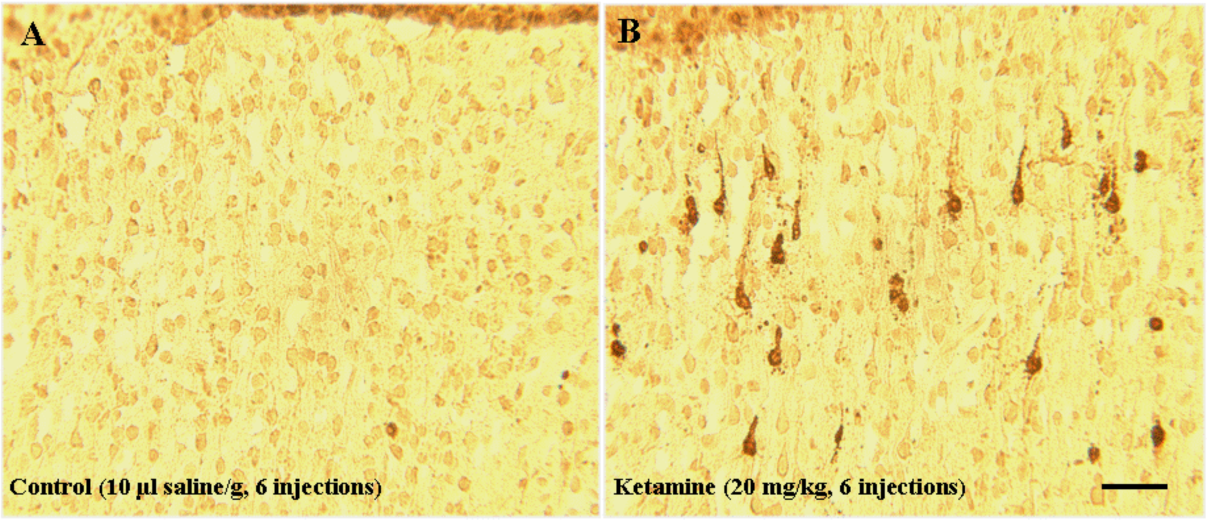

**FIGURE 1.** Ketamine-induced neurodegeneration in PND 7 rats assessed by TUNEL labeling. Representative photographs indicate that TUNEL-positive cells are more numerous in layers II and III of the frontal cortex in the ketamine-treated rat brain (B). Only a few TUNEL-positive cells were observed in the control (saline-treated) rat brain (A). Scale bar = 60 µm. From Shi et al.[16].

On the other hand, in genes that have been down-regulated (17 genes), about one-half are antiapoptotic genes. The overexpression of *Acvr1c* has been shown to suppress the apoptotic effects and *Amigo2* acts as an antiapoptotic factor[18,19]. *Bnip3* encodes cellular proteins that interact with Bcl-2. In cortical cells, cyanide induces a rapid up-regulation of *Bnip3* expression, followed by a caspase-dependent cell death[20]. Down-regulation of *Cd24*, *Cdc2a*, and disruption of the *Rasa1* gene in early embryonic mice induce apoptosis of neuronal cells[21,22]. In neocortical and hippocampal tissues, apoptotic effects can be demonstrated following *Ahr* activation[23] and this gene was found to be up-regulated in our studies. These

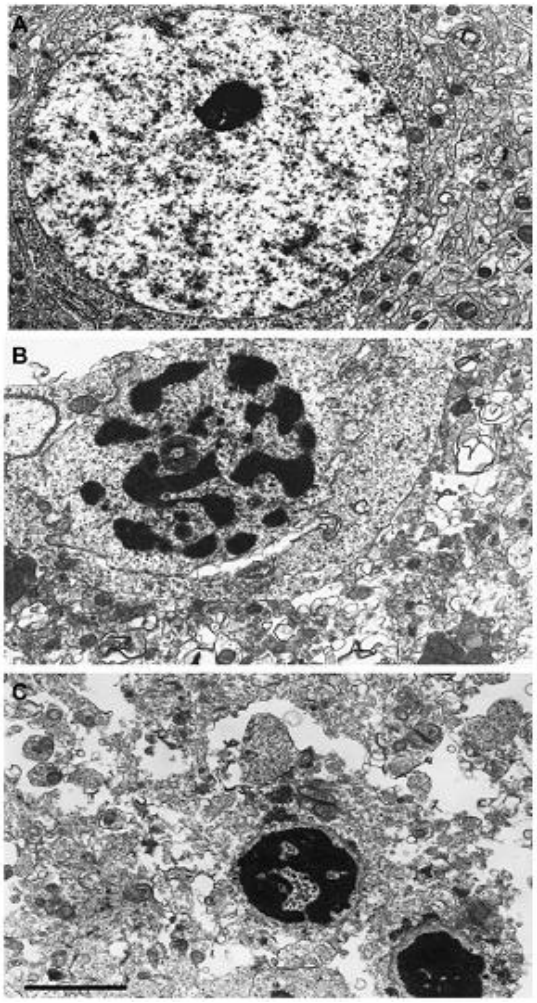

**FIGURE 2.** Electron micrographs (EM) show a normal neuron with intact cytoplasm and nuclear membrane from a PND 7 control rat (A). EMs also show typical nuclear fragmentation (B) and nuclear condensation (C) that represent advanced states of apoptosis in layer II and III of the frontal cortex from ketamine-treated (20 mg/kg × six injections) rat brain. Scale bar = 0.64 µm. From Zou et al.[43].

observations may imply that the frontal cortex is the brain region most vulnerable to ketamine-induced neurotoxicity during development, and the neuronal survival in the early phases of the apoptotic cascades mostly depends on the balance between the pro- and antiapoptotic factors of the apoptosis-related genes.

Our previous data suggest the possible link between the formation of ROS and the regulation of either apoptosis-relevant genes or transcription factors[9,11,24,25]. Several ROS, including nitric oxide and superoxide anion ($O^{-}_2$), have been implicated in anesthetic-induced neuronal death. However, little is known about the signaling pathway that mediates the postulated roles of peroxynitrite (ONOO). In previous *in vitro* studies, general anesthetics, such as ketamine administration, caused a significant up-regulation of nitrotyrosine expression accompanied by enhanced neuronal apoptosis as indicated by cell death detection ELISA and decreased PSA-NCAM expression[26]. Protein tyrosine nitration occurs during many neurodegenerative states and is an important marker of oxidative stress induced by peroxynitrite, and possibly other nitric oxide ($NO^\bullet$)–derived oxidants. The toxicity of $NO^\bullet$ is linked to its

**TABLE 1**
**Apoptosis-Related Genes Identified by GOFFA***

| | Gene Symbols | Gene Names | Fold Change | *p* Value |
|---|---|---|---|---|
| 1 | *Acvr1c* | Activin A receptor, type IC | *–2.1786* | 0 |
| 2 | *Ahr* | Aryl hydrocarbon receptor | *–1.7982* | 0 |
| 3 | *Alms1* | Alstrom syndrome 1 | *–1.6353* | 0 |
| 4 | *Amigo2* | Adhesion molecule with Ig-like domain 2 | *–1.5571* | 0 |
| 5 | *Atp7a* | ATPase, Cu++ transporting, α polypeptide | *–1.5647* | 0.0099 |
| 6 | *Bnip3* | BCL-2/adenovirus E1B 19-kDa interacting protein 3 | *–6.8027* | 0 |
| 7 | *Bub1b* | Budding uninhibited by benzimidazoles 1 homolog, β | *–1.5083* | 0 |
| 8 | *Cd24* | CD24 antigen | *–1.6* | 0 |
| 9 | *Cdc2a* | Cell division cycle 2 homolog A (*S. pombe*) | *–1.5312* | 0 |
| 10 | *Inhba* | Inhibin β-A | *–1.5029* | 0.0001 |
| 11 | *Myc* | Myelocytomatosis oncogene | *–1.9249* | 0 |
| 12 | *Ntf3* | Neurotrophin 3 | *–1.5101* | 0.0063 |
| 13 | *Pak7_predicted* | p21 (CDKN1A) activated kinase 7 (predicted) | *–1.5627* | 0 |
| 14 | *Pdia2_predicted* | Protein disulfide isomerase associated 2 (predicted) | *–1.8522* | 0 |
| 15 | *Rasa1* | RAS p21 protein activator 1 | *–1.8832* | 0 |
| 16 | *Tnfrsf11b* | Tumor necrosis factor receptor superfamily, member 11b | *–1.7039* | 0 |
| 17 | *Unc5c* | Unc-5 homolog C (*C. elegans*) | *–1.5272* | 0 |
| 18 | *Agt* | Angiotensinogen (serpin peptidase inhibitor, clade A, member 8) | *2.9124* | 0 |
| 19 | *Alb* | Albumin | *2.0795* | 0 |
| 20 | *Apoe* | Apolipoprotein E | *1.6286* | 0 |
| 21 | *Bag3* | Bcl-2–associated athanogene 3 | *1.6532* | 0 |
| 22 | *Cebpb* | CCAAT/enhancer binding protein (C/EBP), β | *1.5148* | 0 |
| 23 | *Clu* | Clusterin | *1.703* | 0 |
| 24 | *Cryab* | Crystallin, α B | *1.6146* | 0 |
| 25 | *Gjb6* | Gap junction membrane channel protein β 6 | *2.1345* | 0 |
| 26 | *Hrk* | Harakiri, BCL-2 interacting protein (contains only BH3 domain) | | |
| 27 | *Igfbp3* | Insulin-like growth factor binding protein 3 | *1.5545* | 0 |
| 28 | *Inpp5d* | Inositol polyphosphate-5-phosphatase D | *1.5223* | 0 |
| 29 | *Jun* | Jun oncogene | *1.8866* | 0 |
| 30 | *Mal* | Myelin and lymphocyte protein, T-cell differentiation protein | *2.0125* | 0 |
| 31 | *Rassf5* | Ras association (RalGDS/AF-6) domain family 5 | *1.7735* | 0 |
| 32 | *Txnip* | Thioredoxin interacting protein | *2.3629* | 0 |

* Gene Ontology for Functional Analysis.

ability to combine with superoxide anions ($^{\bullet}O^{-}_{2}$) to form peroxynitrite, an oxidizing free radical that can cause DNA fragmentation and lipid oxidation[27,28]. Recent findings show that peroxynitrite may act as a signaling molecule capable of up-regulating protein tyrosine phosphorylation, which plays an important role in the regulation of cell communication, proliferation, migration, differentiation, and survival[29,30]. In the brain, $NO^{\bullet}$ is produced by neuronal NOS (nNOS) and is believed to be controlled by activation of NMDA receptors[31]. The finding that nNOS is connected to the NMDA receptor via a postsynaptic density protein (PSD95) indicates a direct link between NMDA receptor activation and nNOS stimulation[32]. The interaction of superoxide and $NO^{\bullet}$, which results in the formation of peroxynitrite, can be prevented by targeting either superoxide or $NO^{\bullet}$. The previous study[26] demonstrated that pharmacological manipulation of NOS by 7-nitroindazole provides protection against ketamine-induced neuronal loss. Importantly, no protective effect was observed with cultures cotreated with ketamine and 7-nitroindazole, when 7-nitroindazole was omitted during ketamine washout. Primary neuronal cell cultures are thought to mainly contain nNOS. Therefore, 7-nitroindazole was used in this study since it has been reported to be a selective nNOS inhibitor. It has been demonstrated that pretreatment with 7-nitroindazole blocks methamphetamine (METH)–induced dopaminergic neurotoxicity in mice[33] and selective nNOS knock-out mice are resistant to METH-induced dopaminergic neurotoxicity[34]. In addition to 7-nitroindazole, several other NOS inhibitors and iron porphyrin compounds have been utilized, such as FeTPPS and FeTMPyP, which efficiently degrade peroxynitrite to nitrate under physiological conditions. It appears, however, that 7-nitroindazole is the most selective nNOS inhibitor[35,36,37].

The effects of antioxidants indicate the role of ROS in anesthetic-induced neuronal death, but it is noted that no direct measurement of ROS has been made in anesthetic-treated neurons, which is essential to confirm the involvement of ROS. Additional experiments to address this issue are necessary.

## ANESTHETIC-INDUCED UP-REGULATION OF THE NR1 SUBUNIT AND NEURODEGENERATION

It is important to indicate that up-regulation of the NR1 subunit of the NMDA receptor is an important first step in the pathway to anesthetic-induced neurotoxicity. These up-regulated calcium channel receptors are vulnerable to endogenous glutamate concentrations within the tissues after ketamine washout. In our previous studies[8,11], to test whether the administration of antisense oligonucleotides (ODNs) targeted to the NR1 NMDA receptor subunit blocks steady-state protein levels, an antisense ODN-targeting exon was used. Coadministration of antisense ODN specifically prevented NR1 up-regulation, blocked the reduction of PSA-NCAM expression induced by ketamine, and protected neurons from apoptosis.

Of particular interest to the data at hand are the possible mechanisms by which anesthetics, such as ketamine, could up-regulate NMDA receptors. Surprisingly, there is not an abundance of literature concerning this issue, but recently it has been demonstrated that the distal region of the NR1 promoter contains an active NF-κB site, which is developmentally regulated and appears to bind Sp3/Sp1 somewhat better than the NF-κB subunits[38]. The NMDA receptor NR1 subunit is widely distributed throughout the brain and is the fundamental subunit necessary for NMDA channel function. Our previous study[11] demonstrated that ketamine produced a significant up-regulation in NR1 protein expression. This result was consistent with literature demonstrating that treatment with NMDA antagonists produces an up-regulation of the NMDA receptor complex as measured by an increase in *B*max of NMDA receptor binding sites[39,40].

The transcription factor NF-κB is known to respond to changes in the redox state of the cytoplasm and has been shown to translocate in response to NMDA-induced cellular stress[41]. NF-κB is normally sequestered in the cytoplasm, bound to the regulatory IκB proteins. The net result is the release of the NF-κB dimer, which is then free to translocate into the nucleus.

NF-κB translocation appears to be a necessary step in cell death induced by PCP[25], cyanide, and excitotoxic stimuli[42]. In our previous studies, ketamine produced a remarkable increase in translocation of NF-κB into the nucleus. The protection against cortical neuronal cell death and decreased PSA-NCAM by a peptide inhibitor of NF-κB translocation, SN-50, suggested that there was a causal relationship between these effects. There is evidence in the literature suggesting that the transcriptional regulation of target genes by NF-κB is tissue specific and possibly gene specific within a given cell type. The ability of SN-50 to prevent ketamine-induced cell death demonstrated that NF-κB is crucial to those processes. However, whether anesthetic-induced NF-κB translocation is specifically responsible for apoptotic or necrotic pathways observed in anesthetic studies is still unknown. Resolution of this question will require additional experiments.

In line with previous morphological and biochemical findings on anesthetic agents, such as ketamine, our microarray study[16] identified disruptions in glutamate receptor signaling, synaptic long-term potentiation, PTEN (phosphatase and tensin homolog deleted on chromosome 10) signaling, pyrimidine metabolism, circadian rhythm signaling, etc. The gene expression of the NMDA receptor subunit gene, *Grin1* (NR1), was significantly up-regulated in ketamine-treated rat pups as detected in microarray experiments and subsequently confirmed with TaqMan analyses. These data indicate that ketamine-induced pathological change is closely associated with the remarkable up-regulation of the NMDA NR1 subunit mRNA.

It is possible that increased expression of *Grin1* (NR1) was accompanied by an altered expression of other glutamate receptor subunits. Our microarray analyses and Q-PCR data[16] revealed an increase in *Grin2a* (NR2A; 1.5-fold) and *Grin2c* (NR2C; 1.7-fold), but no significant effects were observed in *Grin2b* (NR2B) or *Grin2d* (NR2D). Our findings are consistent with those of previous *in situ* hybridization and immunoblotting data that demonstrated a compensatory up-regulation of NMDA-R1 and NMDA-R2 receptors following prolonged exposure to NMDA receptor antagonists[7,9,43].

## SUMMARY

In summary, Fig. 3 illustrates a potential model of anesthetic-induced (i.e., ketamine) neuronal cell death in the developing brain. Excessive activation of up-regulated NMDA receptors results in a calcium overload that exceeds the buffering capacity of the mitochondria and interferes with electron transport in a manner that results in an elevated production of ROS, and the dissociation of some transcription proteins, such as NF-κB, and their transport into the nucleus. In the nucleus, these transcription factors bind to several DNA sequences of several known genes. The consequence of this binding is not completely understood, but the loss of the balance of pro- and antiapoptotic genes is apparent; the diminished formation of antiapoptotic heterodimers in favor of proapoptotic homodimers. These homodimers are thought to create mitochondrial membrane pores through which cytochrome c can leak into the cytoplasm where it can activate caspases that play a critical role in the ultimate demise of the neuron. As shown above (text), several recent studies using antisense ODNs targeting specific NMDA receptor subunits, or blockers of oxidative stress, such as L-carnitine, the superoxide dismutase mimetic, M40403, and the NOS inhibitor, 7-nitroindazole, have indicated that reduction of oxidative stress may protect the developing animal from anesthetic-induced brain cell death.

## FUTURE DIRECTIONS AND CHALLENGES

Our findings suggest that anesthetic-induced cell death in the rat brain seems to be mainly apoptotic in nature. Necrosis is not observed in the anesthetic-treated rat brain, although necrosis is a consequence in the anesthetic-treated nonhuman primate[9]. The mechanisms by which different consequences are caused in different species warrant further studies. Although repeated experiments confirmed the role of up-regulated NR1 in anesthetic-induced neurodegeration, more experiments are needed to validate the hypothesis. For example, direct evidence showing that up-regulated NR1 causes global calcium influx in neurons is necessary. Moreover, the roles of ROS in anesthetic-induced neurodegeneration need further validation.

## DISCLAIMER

This document has been reviewed in accordance with U.S. Food and Drug Administration (FDA) policy and approved for publication. Approval does not signify that the contents necessarily reflect the position or opinions of the FDA nor does mention of trade names or commercial products constitute endorsement

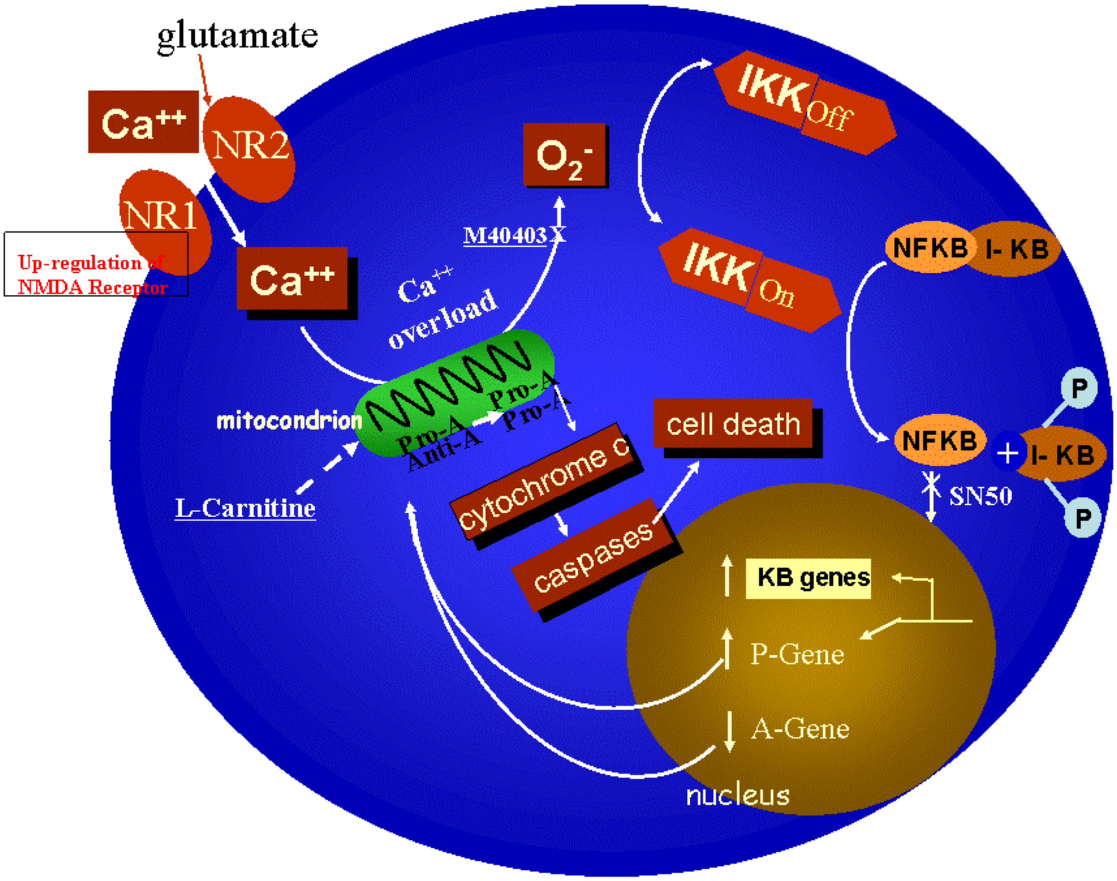

**FIGURE 3.** The cartoon illustrating the working model of anesthetic-induced (e.g., ketamine) neuronal cell death and potential protection mechanisms.

or recommendation for use. The findings and conclusions in this report are those of the authors and do not necessarily represent the views of the FDA.
